# Advancements in curcuminoid formulations: An update on bioavailability enhancement strategies curcuminoid bioavailability and formulations

**DOI:** 10.1515/biol-2025-1112

**Published:** 2025-07-18

**Authors:** Alexander Darmonkow, Zoë E. M. Rowe, Scott V. Harding

**Affiliations:** Faculty of Medicine, Memorial University, St. John’s, NL, Canada; Department of Biochemistry, Faculty of Science, Memorial University, St. John’s, NL, Canada

**Keywords:** curcumin, bioavailability, dietary supplement, functional food

## Abstract

Here, we provide a current review of strategies aimed at improving the bioavailability of curcuminoids, a group of compounds with therapeutic potential. This review discusses formulations from the traditional supplementation approaches to the innovative methods to enhance solubility and bioavailability, including cyclodextrins and hydrophilic carriers. Additionally, colloidal delivery strategies, such as micelles, liposomes, solid lipid particles, and emulsions, are examined as promising vehicles for curcuminoid delivery. The review underscores the importance of clinical trials in assessing the efficacy of these formulations and highlights a pivotal yet frequently neglected factor in curcuminoid research: the differentiation between total and free curcuminoid quantification. In summary, this concise review evaluates existing curcuminoid formulations and explores innovative approaches to improve their bioavailability.

## 1 Introduction

Turmeric (*Curcuma longa)*, is a popular spice, medicinal herb, and pigment originating from Southeast Asia and India, which now has a large and rapidly growing global market [1]. In addition to its popularity in food preparation, turmeric’s long history and growing popularity can be attributed to a wide range of reported medicinal benefits, many of which have now been extensively studied. The pharmacological effects of turmeric are due to its main group of bioactive compounds, curcuminoids, and lipophilic polyphenols which include curcumin and related compounds dimethoxycurcumin and bisdemethoxycurcumin [2,3]. The curcuminoids (and their derivatives) have been reported to exhibit numerous therapeutic effects, including anti-inflammatory, antioxidant, anti-tumor, antimicrobial, neuroprotective, and cardioprotective effects, and therefore have received significant attention as a therapy for a wide variety of diseases [2]. Despite the exhaustive list of potential therapeutic effects, a commonly cited limitation of curcumin supplementation is its low bioavailability due to chemical instability during digestion and its pharmacokinetics [4]. This has led to recent research efforts to develop formulations of curcumin that enhance its bioavailability by way of increased free curcuminoid or curcuminoid metabolite levels, and, in turn, increasing the curcumin therapeutic effect [5]. This review introduces the curcuminoids and their chemical/metabolic by-products, briefly comparing their therapeutic potential, then examines current formulations that show promise for unlocking the full potential of this herbal treatment.

## 2 Chemical structure

**Figure 1:**
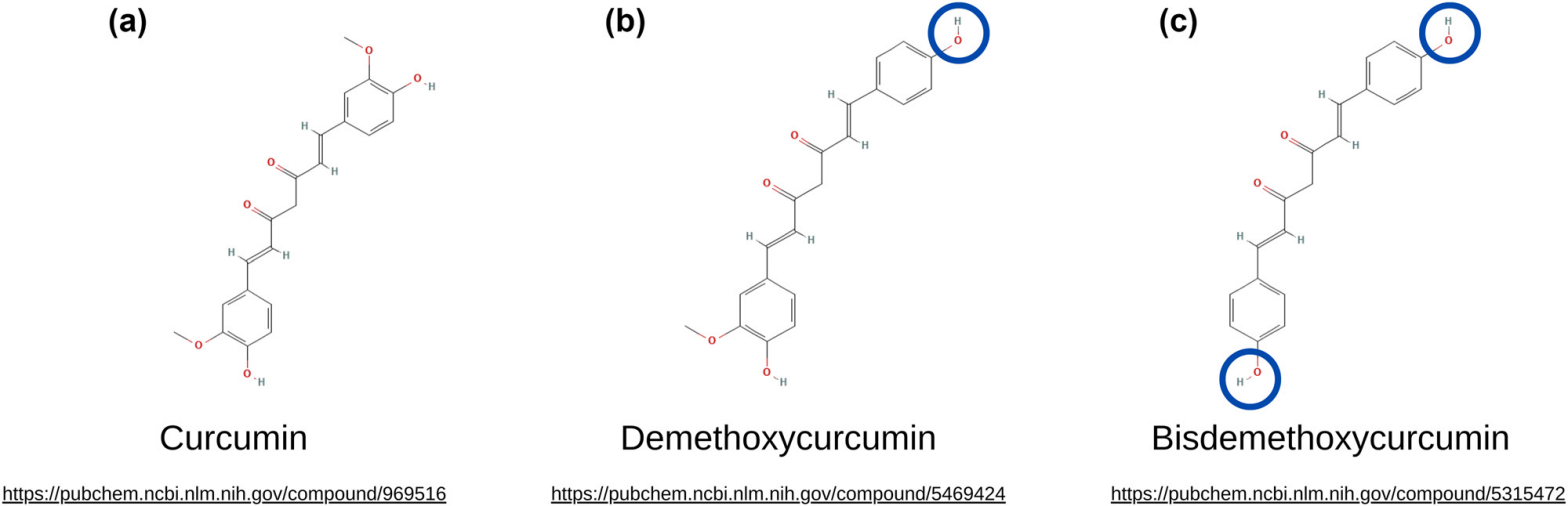
Chemical structures of the three major curcuminoids found in turmeric. Structures were extracted from PubChem [20]: (a) Curcumin, PubChem compound ID 969516, (b) Demethoxycurcumin, PubChem ID 5469424, (c) Bisdemethoxycurcumin, PubChem ID 5315472. Structural differences between curcumin and demethoxycurcumin and bisdemethoxycurcumin are noted with blue circles on the differing reactive functional groups.

Turmeric contains between 2 and 9% total curcuminoids, by weight which further breaks down to approximately 75% curcumin, 20% demethoxycurcumin, and 5% bisdemethoxycurcumin [6]. The lipophilic polyphenol structure of the curcuminoids (Figure 1) – two benzomethoxy rings joined by an unsaturated carbon chain – gives curcuminoids very low water solubility at neutral or acidic pH, but good solubility in solvents such as methanol, ethanol, and acetone [2]. Curcumins structurally symmetrical hydroxyl groups contribute to their hydrophobicity resulting in relatively poor solubility (0.6 µg/mL) and therefore low bioavailability in the digestive tract [7]. The unsaturated carbon chain of the curcuminoids contains a diketone moiety, which exhibits keto-enol tautomerism that favors the enol form when dissolved in solution [8]. This moiety can act as a metal chelator and composes one of three primary reactive sites on curcuminoids, including a nucleophile acceptor on the carbon chain double bond, and a hydrogen atom donor on the aromatic ring’s hydroxyl group [1].

## 3 Products and metabolites

Curcumin undergoes chemical degradation at physiological pH, which increases in rate as pH increases [6]. The degradation is due to an alkaline hydrolysis reaction, which results in products such as ferulic acid, vanillin, ferulaldehyde, and feruloyl methane [9]. Autooxidative cyclization, an alternate and less recognized pathway, produces a deoxygenated dicyclopentadiene an alternate, but less recognized pathway, is that of autoxidative cyclization which produces a deoxygenated dicyclopentadiene [4,10].

Ferulic acid and vanillin show the most therapeutic potential of the chemical degradants and are therefore the most studied, supported by numerous *in vitro* studies showing antioxidant activity, anti-inflammatory activity, and anticancer activity in prostate and colorectal cell lines, respectively [8,11]. However, cancer cell line studies show that the effect of the degradation products is reduced in comparison to the parent curcuminoid compounds [8]. There are very few *in vivo* studies that examine the effect of either curcuminoid parent compounds or their degradation products [12–14].

One double-blind, randomized controlled trial (RCT) by Bumrungpert et al. [15] found ferulic acid to have a significant effect versus placebo in improving lipid profiles and oxidative stress levels in hyperlipidemic subjects. This study shows promise for the degradation products of curcuminoids as interventional therapies, which could also have other desirable pharmacological effects *in vivo*. In summary, it appears the chemical degradation products of the curcuminoids, especially ferulic acid and vanillin, could be contributing to some of the observed pharmacological effects of curcuminoids both *in vitro* and *in vivo*, though not as powerful as free curcuminoids themselves. Further inquiry into their greatest application is a potential future research pathway.

The metabolic processing of curcuminoids leads to the formation of distinct products, with the possibility of generating chemical degradants *in vivo*. Once absorbed, curcuminoids follow either of two primary metabolic pathways, resulting in Phase I reduction or Phase II conjugation metabolites. Phase I metabolites encompass dihydrocurcumin, tetrahydrocurcumin, and hexahydrocurcumin, while Phase II metabolites include curcumin sulfate and curcumin glucuronide [16]. Notably, in both rats and humans, phase II glucuronide and sulfate conjugates are the most prevalent metabolites in the bloodstream. As discussed earlier, the detection of free curcuminoids is minimal [8]. Unfortunately, research suggests that these available metabolites exhibit significantly lower therapeutic potential compared to curcuminoids themselves, particularly in terms of *in vitro* chemopreventive effects [17,18].

In a separate *in vitro* study, Choudhury et al. [19] investigated the antioxidant activity of two curcumin glucuronides (monoglucuronide and diglucuronide) in comparison to curcumin, utilizing free radical DPPH scavenging and an oxygen radical absorbance capacity assay. The results indicated a ten-fold reduction in antioxidant activity for monoglucuronide, while the antioxidant activity of diglucuronide was entirely insignificant when compared to curcumin. In summary, existing literature suggests that these metabolic products also exhibit diminished therapeutic potential compared to free curcuminoids.

## 4 Signaling pathways

Curcumin is a potent bifunctional antioxidant that directly and indirectly scavenges reactive oxygen species and there is great potential in minimizing age-related cellular damage. In addition to the presence of phenolic groups that serve as hydrogen donors, curcumin can also activate the nuclear factor-erythroid-2-related factor 2 (Nrf2)/antioxidant response element (ARE) pathway [21]. Nrf2 is an important transcription factor that protects cells from endogenous and exogenous stressors. Under oxidative stress, Kelch-like ECH-associated protein 1 (Keap1), cytoplasmic protein, senses redox imbalances and dissociates from Nrf2. Subsequently, Nrf2 translocates to the nucleus and activates the transcription of several antioxidant enzymes such as thioredoxin reductase, Hsp70, heme oxygenase, and sirtuins [22]. The enzymes are known to inhibit oxidative stress and serve as a major defense system against cellular stress and oxidative damage [23]. The exact mechanism of curcumin interaction with the Nrf2/ARE pathway remains unclear, and further investigation may provide a clear understanding of curcumin health benefits.

## 5 Absorption and excretion

Curcumin absorption is generally characterized by poor bioavailability which can be explained, in part, by its instability in aqueous environments resulting in rapid hydrolysis and low absorption rates at intestinal pH. Additionally, a significant portion of curcumin is metabolized in the intestines and liver, reducing the amount of curcumin in its active form. These factors, along with its hydrophobic nature and fast excretion in the feces, contribute to low serum concentrations. In a bolus dose-escalation study conducted by Lao et al. [24], using a standardized powder extract (95% curcuminoids) and were unable to detect any curcumin in participant serum samples at lower physiological doses. They were able to detect serum curcumin in the two highest dose levels provided; 10,000 and 12,000 mg but even at the largest dose detected levels were still quite low, reaching a maximum of 57.6 ng/ml 2 hours after consumption of 12,000 mg. However, the absence of any severe adverse effects – dose-independent diarrhea was observed – demonstrates a safe tolerance to large doses of curcuminoids.

Similarly, Vareed et al. [25] administered 10 and 12 g doses of a standardized extract, employing high-performance liquid chromatography (HPLC) for analysis. They detected free curcumin in only one out of twelve participants’ serum samples, while curcumin glucuronides and sulfates were detected in all cases. Additionally, Holder et al. [26], using radioactive labeling, observed that a significant portion of a standard extract curcumin dose is excreted in rat feces. The major biliary metabolites included glucuronides of tetrahydrocurcumin and hexahydrocurcumin, along with chemical degradants such as dihydroferulic acid and trace ferulic acid. The almost negligible levels of free curcumin in the serum, coupled with detectable metabolite levels (glucuronides and sulfates) and a substantial concentration in the feces, collectively affirm the widely accepted notion of low curcuminoid bioavailability. The majority of a curcumin dose undergoes either metabolism or excretion very quickly following digestion and absorption.

## 6 Formulations

Curcumin and its analogues exhibit low solubility in physiological conditions and face challenges in absorption within the gastrointestinal tract. Moreover, the absorbed curcumin undergoes rapid metabolism, yielding comparatively fewer active products and displaying chemical instability. These inherent characteristics of curcuminoid compounds, coupled with their extensive therapeutic potential, have prompted the development of numerous technologies and formulations aimed at enhancing the bioavailability of orally administered curcuminoids.

One of the initial and widely adopted strategies to enhance the bioavailability of curcumin involves its co-administration with piperine, an alkaloid found in black pepper. Piperine exhibits the ability to inhibit hepatic/intestinal glucuronidation and *p*-glycoprotein-mediated intestinal excretion [27]. In a human study, the administration of a 2 g dose of curcumin with 20 mg piperine resulted in a significant increase in serum levels within the first hour, boosting bioavailability by 2,000% compared to curcumin alone [27]. Recent research further supports the efficacy of this formulation in a clinical context. Panahi et al. [28] conducted a study involving 70 non-alcoholic fatty liver disease (NAFLD) patients who were randomly assigned to receive either a 500 mg curcuminoid with 5 mg piperine or a placebo. The piperine group exhibited significant decreases in serum alanine aminotransferase, aspartate aminotransferases, and cholesterol, along with a notable improvement in NAFLD severity. Tabanelli et al. [29] used a different approach to achieve higher curcumin bioavailability, combining curcuminoids with non-curcuminoid components of turmeric, such as essential oils and/or powder. These formulations, containing lipophilic turmeromes, have been shown to enhance curcuminoid absorption and inhibit p-glycoprotein, akin to the effects of piperine [29]. In human volunteers, a patented formulation known as BCM-95®CG (Biocurcumax™) demonstrated a 6.93-fold increase in bioavailability compared to standard curcumin powder and a 6.3-fold increase compared to a piperine formulation [30]. These findings underscore the effectiveness of combining piperine or turmeric components with curcuminoids in significantly increasing bioavailability and subsequently, demonstrating clinical significance.

While the above-mentioned approaches aim to improve the absorption of curcuminoids by influencing the gastrointestinal tract, more innovative formulations leverage adsorptive properties and/or inclusion complexes to boost solubility and consequently enhance the bioavailability of curcuminoids. Cyclodextrins, such as connected cyclic oligosaccharides with hydrophilic glucose units on the exterior, exemplify this concept. These cyclodextrins can form complexes with fat-soluble compounds within an interior lipophilic cavity [29]. In a study conducted by Purpura et al. [31], the bioavailability of curcuminoids from a g-cyclodextrin formulation was compared to that of a standardized extract, phytosome, and turmeric essential oil formulation in healthy volunteers. The cyclodextrin formulation demonstrated the most significant increase in total curcuminoid bioavailability, elevating plasma levels by 39-fold compared to the standard curcuminoid extract. Another formulation within this category involves hydrophilic carriers consisting of polyvinyl pyrrolidone (PVP), cellulose derivatives, antioxidants, and small amounts of fat, which disperse curcuminoids to overcome low solubility in physiological conditions [5]. Indeed, this particular formulation has been directly proven to enhance bioavailability over all other discussed formulations. In a double-blind randomized controlled trial conducted by Jäger et al. [32], which compared the effects of multiple formulations on curcuminoid blood levels using HPLC and mass spectrometry, the hydrophilic carrier formulation exhibited significantly increased absorption compared to a phytosome formula (5.8-fold), an essential oil formula (34.9-fold), and a standard extract (45.9-fold). When examining clinical studies for a g-cyclodextrin formulation patent (Cavacurmin®) and a hydrophilic carrier patent (Curcuwin®), a remarkable 85-fold and 136-fold increase in total curcumin, respectively, was observed [29]. Collectively, this body of research underscores the effectiveness of the hydrophilic carrier formulation in enhancing curcumin bioavailability in humans.

## 7 Enhancement strategies

In addition to the previously discussed formulations, researchers are exploring various colloidal delivery strategies to enhance the bioavailability of curcuminoids. These strategies involve the encapsulation of curcuminoids in edible micro- or nanoparticles within micelles, liposomes, solid lipid particles, and emulsions, among others [33]. Encapsulation techniques involve entrapping bioactive substances (i.e., polyphenols) within small particles dispersed in water. This delivery system isolates the particles from the environment and protects them from degradation within the digestive tract [33]. These different colloidal transport mediums naturally find varying applications, each suitable or less fitting as therapeutic interventions. Overall, however, they constitute highly effective formulations for curcuminoids.

In a human RCT, Fança-Berthon et al. [16] evaluated the total absorption of curcuminoids using a dried colloidal suspension (composed of standard turmeric extract, quillaja extract, sunflower oil, and acacia gum), alongside a standardized turmeric extract, liquid micelle, piperine, and phytosome formulation. Employing HPLC and mass spectrometry, the study revealed that both the dried colloidal suspension and the micellar preparation produced the highest levels of total curcuminoids. After dose-normalization, both formulations resulted in significantly higher levels compared to the standardized extract, with the dried colloidal suspension exhibiting a notable increase relative to the phytosome and piperine formulation as well [16].

However, a noteworthy observation emerged when the researchers specifically compared the levels of unconjugated plasma curcuminoids; in this aspect, no significant differences between the formulations were observed. This underscores a crucial consideration in studies focusing on curcuminoid bioavailability and formulation.

## 8 Considerations and conclusions

As observed earlier, numerous studies gauge blood or plasma levels of total curcuminoids to assess the impact of different formulations on bioavailability. However, certain studies, including some referenced in this article, treat plasma samples with β-glucuronidase and sulfatase enzymes during analysis, enabling the measurement of these conjugates as “free” curcuminoids [34]. Under this methodology, the term “total curcuminoids” encompasses not only free curcuminoids but also the conjugate products resulting from curcumin metabolism. It is noteworthy that these conjugates generally exhibit lower bioactivity compared to free curcuminoids. Consequently, an increase in total curcuminoid plasma levels may not necessarily translate to heightened free curcuminoid bioavailability and clinical efficacy.

In their methodology, Fança-Berthon et al. [16] explicitly specified the extraction of plasma with or without deconjugation, subsequently reporting results for both extraction methods. However, as highlighted by Stohs et al. [34], numerous studies omit providing such clarity in their information. This omission can obscure the genuine value of a specific formulation, as it might seem to significantly enhance bioavailability but ultimately provide limited clinical value due to an abundance of conjugated, rather than free, curcuminoids. This aspect introduces a considerable “grey area” in studies on curcumin formulations. Future research on bioavailability should, therefore, distinctly distinguish between total and free curcuminoid measurements to facilitate a more precise evaluation of formulations. Even with a clear description of curcuminoid measurement, a clinical trial would still be necessary to assess the therapeutic potential of a formulation demonstrating increased free curcuminoid bioavailability. Despite the demonstrated potential, it appears that straightforward yet thorough research remains the primary constraint in fully understanding the effects of curcuminoids.

Other non-nutritive components of foods have a similar research limitation, very rapid conjugation and excretion from the body. This creates a very difficult situation for researchers looking to examine the biological and physiological effects of compounds like curcuminoids. Opportunities exist with the advancement of computational methodologies for those researchers looking at direct interactions with enzymes or other compounds in the body. Advanced pre-clinical or clinical techniques that use stably labelled isotopes also offer a potential advantage over the methods used to date for determining the function and fate of curcuminoids in humans. Hopefully, this research will continue to advance our understanding of these compounds and provide consumers with confidence in their purchasing of the nutraceutical and supplement formulations of these turmeric-derived products.
